# Facebook Advertising Across an Engagement Spectrum: A Case Example for Public Health Communication

**DOI:** 10.2196/publichealth.5623

**Published:** 2016-05-30

**Authors:** Tevah Platt, Jodyn Platt, Daniel B Thiel, Sharon L. R Kardia

**Affiliations:** ^1^ School of Public Health Department of Epidemiology University of Michigan Ann Arbor, MI United States; ^2^ Medical School Department of Learning Health Sciences University of Michigan Ann Arbor, MI United States; ^3^ School of Public Health Department of Health Management and Policy University of Michigan Ann Arbor, MI United States

**Keywords:** Internet, facebook, social media, facebook advertising campaign, social media engagement, health communication, social networking, biobanking, neonatal screening, infant, newborn screening

## Abstract

**Background:**

The interpersonal, dialogic features of social networking sites have untapped potential for public health communication. We ran a Facebook advertising campaign to raise statewide awareness of Michigan’s newborn screening and biobanking programs.

**Objective:**

We ran a Facebook advertising campaign to stimulate public engagement on the complex and sensitive issue of Michigan’s newborn screening and biobank programs.

**Methods:**

We ran an 11-week, US $15,000 Facebook advertising campaign engaging Michigan Facebook users aged 18-64 years about the state’s newborn screening and population biobank programs, and we used a novel “engagement spectrum” framework to contextualize and evaluate engagement outcomes ranging from observation to multi-way conversation.

**Results:**

The campaign reached 1.88 million Facebook users, yielding a range of engagement outcomes across ad sets that varied by objective, content, budget, duration, and bid type. Ad sets yielded 9009 page likes (US $4125), 15,958 website clicks (US $5578), and 12,909 complete video views to 100% (US $3750). “Boosted posts” yielded 528 comments and 35,966 page post engagements (US $1500). Overall, the campaign led to 452 shares and 642 comments, including 176 discussing newborn screening and biobanking.

**Conclusions:**

Facebook advertising campaigns can efficiently reach large populations and achieve a range of engagement outcomes by diversifying ad types, bid types, and content. This campaign provided a population-based approach to communication that also increased transparency on a sensitive and complex topic by creating a forum for multi-way interaction.

## Introduction

Social networking sites have the potential to modernize core public health functions, including the delivery of essential services [1,2]. They can also be used to focus on specific populations, including some that are vulnerable and hard to reach [2]. Fostering ongoing, multi-way communication, these sites encourage new thinking about the role the public can and should play in public health. As tools for establishing and maintaining new virtual relationships and communities, they would also be well suited to accompany national health initiatives such as the Affordable Care Act, precision medicine, and learning health systems, perhaps catalyzing a new kind of personalized public health.

With more than a billion users, Facebook is the most popular social networking site in the United States and the world [2,3]. Facebook ads can reach and engage large, finely specified populations at relatively low cost [4-6]. Obtaining Web-based health information is a common practice among Internet users [7,8]. In addition, although some have found that samples drawn from social networking sites tend to overrepresent females and young adults [8], factors that do not significantly affect participation in social networking sites include household income level, race or ethnicity, and geographic location [9], suggesting Facebook’s broad potential to reach a large and diverse audience.

In this study, we present an example of a recent public health Facebook advertising campaign, whose goal was to engage Michigan citizens on the topic of the state’s newborn screening and large population biobanking programs. The results from this low-cost, short-duration campaign demonstrate potential for public health communicators to move beyond passive approaches to reach communications goals across a spectrum of engagement levels that include active participation by the public. The focus of the Facebook advertising campaign presented here is the Michigan BioTrust for Health (BioTrust), a population-based biobank run by the state’s health department that functionally links public health practices and the research enterprise. The BioTrust’s biobank is composed of residual dried bloodspots retained after newborn screening that can be linked to public health registries and other health information sources.

Biobanks such as this have raised legal and ethical questions around the limits of informed consent and the role of participants in unspecified research [10]. An ongoing critique of the open-ended storage and unspecified use is that ensuring participants have made an informed decision is nearly impossible, and lack of transparency has undermined trust in some large population biobanks. Lawsuits driven by privacy concerns in Texas and Minnesota led to the destruction of dried bloodspots stored by those state health departments [11]. Although efforts are underway to address these issues procedurally (eg, proposed revisions to the Common Rule announced in 2015 would require broad consent for or notification of secondary research), another key approach is to improve education and communication to maintain respectful participant partnerships and ongoing public engagement [12,13]. Social networking sites open possibilities for communication to occur organically, an outcome unlikely to be sparked by one-time notification or consent.

Michigan BioTrust for Health markets a biobank of more than 4 million deidentified dried bloodspots to health researchers. However, its participants are largely unaware of their participation. Less than half of Michiganders are aware of newborn screening and less than 5% are aware of the BioTrust [14]. Raising statewide awareness of Michigan’s biobank is a challenging undertaking. The participant group is large and transient; the message is sensitive and complex [4].

Various models have been used to describe and evaluate a hierarchy of engagement outcomes in social media [15-19]. Preece and Schneiderman [18], for example, propose a generalized “Reader to Leader framework” that describes engagement in terms of the way in which people may become active readers, contributors, collaborators, or leaders on Web-based social media forums. In a systematic review, Hrastinski [19] iteratively develops a six-stage classification scheme with definitions of participation ranging from “accessing e-learning environments” to taking part in dialogue. Neiger et al distinguish low (eg, likes), medium (eg, content sharing), and high social media engagement (eg, partnership, online action) in the context of Twitter [16,20].

**Figure 1:**
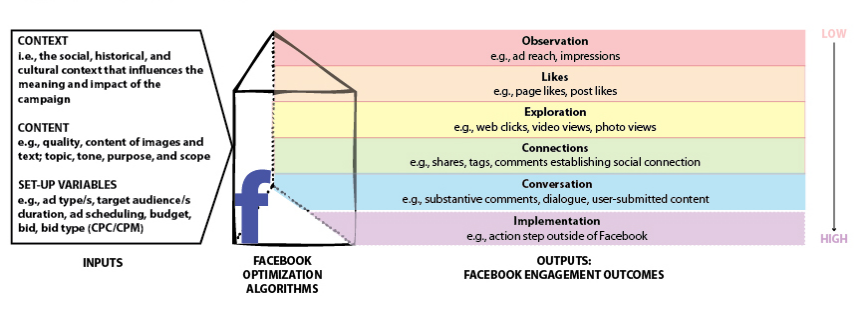
A spectrum of Facebook engagement outcomes.

Each of these frameworks highlights the potential for social media sites to achieve various modes of participation. Drawing heavily on the evaluation hierarchy described by Neiger et al, we developed a framework for examining a spectrum of Facebook engagement outcomes (Figure 1). We consider as inputs (1) context, (eg, the social, political, and cultural context in which the campaign occurs), (2) content (eg, quality and content of ads), and (3) setup variables (eg, ad type, target audience, budget, and so on). These are then processed through Facebook’s optimization algorithms or the “prism” that leads to Facebook engagement. These can be classified along a spectrum ranging from low to high levels of engagement, wherein we have included, where possible, metrics specific to the Facebook advertising platform.

In the lower engagement range, Facebook users are observing (red), “liking” (orange), and exploring content by clicking on links to photos, videos, and websites (yellow). At these levels, a campaign’s goals include reaching the targeted audience, establishing a community of followers to receive ongoing content, and delivering information. In the higher engagement range, Facebook users are establishing connections by sharing posts or commenting (green) and conversing on the Facebook platform (blue). Goals at the higher levels of engagement may include stimulating peer-to-peer content sharing, identifying and addressing questions and concerns from the public, or establishing partnerships. The highest level of engagement, which we have delineated as “implementation” (purple), is an action step taken outside of the Facebook domain (eg, attending an event, or enrolling in a research study.)

This Facebook ad campaign took a population-based approach to achieving engagement across the spectrum by creating a forum for multi-way interaction. In this paper, we present and apply the spectrum of Facebook engagement outcomes framework to describe the campaign. We then consider the implications of these results for public health communicators to move beyond passive approaches to achieve a range of communications goals.

## Methods

In the spring of 2015 the University of Michigan’s Life Sciences and Society Program ran an 11-week Facebook advertising campaign, with a US $15,000 budget, to raise awareness and understanding of the state’s newborn screening and biobanking programs among Michigan Facebook users aged 18-64 years.

### Ad Content Preparation

Visual content for the ads included a graphic image of the state of Michigan, 2 animated, ~1-minute videos (using Sparkol VideoScribe software), and 35 photographs of Michigan biobank participants and parents. We took these photos in organized sessions in which we educated 80 individuals (biobank participants or parents of children with bloodspots in the biobank) about Michigan’s newborn screening and biobank programs, and then asked them to create posters to share their thoughts. We photographed participants holding up posters they created from the suggested prompts: “Today I learned...;” “I hope...;” “I was spotted in [place of birth];” and “I’ll share what I learned because....” Photographed participants were compensated with a US $25 cash card incentive and signed photo release forms that included permission to use images for educational, academic, and research purposes.

We also photographed participants pointing to Michigan locations on their hands, a familiar gesture in this mitten-shaped state. We used the tagline “Where were you spotted?” throughout the campaign, highlighting Michigan locations and soliciting comments from Facebook users indicating where in the state they or their children were born.

We used the University of Michigan School of Public Health logo as our profile image (which appears in all ads) to help establish credibility and to distinguish ourselves from the state health department and the BioTrust.

### Ad Types and Ad Sets

Ad content is uploaded onto Facebook’s Ads Manager. Using the Ads Manager interface, we selected the objective (ie, “ad type”), budget (total amount spent on an ad type), and “bid type,” which determines how costs are incurred (ie, on a per-click or per-viewer basis) and how Facebook will promote the ad (Figure 1). Ad types correspond with advertising objectives defined by Facebook, including page likes, event responses, and app installs. Ad design features such as character limit, image size, and call-to-action buttons vary slightly across ad types. The ad type designation also affects how Facebook optimizes delivery of ads for maximum impact; for example, the type of ad that maximizes “video views” is more likely to be delivered to Facebook users who watch videos.

**Figure 2:**
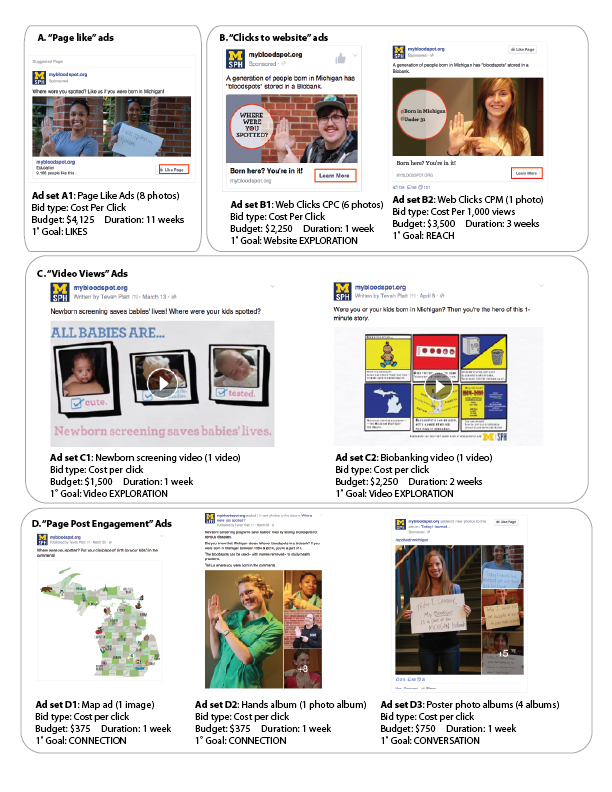
Facebook ad set examples. All ads targeted Michigan residents aged 18-64 years. Ad set A1 included an additional audience of Michiganders aged 18-64 years interested in parenting. Ad set identifiers (A1-D3) correspond with Table 1 and references within the manuscript.

As shown in Figure 2, our campaign used 4 ad types: (1) A, “page likes,” which encouraged users to like our Facebook page; (2) B, “clicks to website”; (3) C, “video views”; and (4) D, “page post engagement,” boosting interactions such as comments, shares, and photo views. The campaign included a total of 8 ad sets that varied by budget, schedule, ad type, and bid type. Ad sets had “lifetime budgets” equally apportioned across their target audiences. All ads ran at the bid price automated by Facebook and incurred cost based on clicks (cost per click, CPC), except for ad set B2, which was set to a bid of US $2 per thousand impressions (cost per 1000 impressions, CPM).

Although no ad type aimed to achieve a single engagement goal exclusively, each ad set was designed to hit 1-2 primary engagement goals, noted in Figure 2. For example, “reach” was the primary goal of ad set B2, with inputs that prioritized reach over clicks. Likes were the main objective of ad set A. We designed ad sets B1-B2, C1-C2, and D3 to encourage exploration of our website, videos, and photo albums showing biobank participant perspectives. By incorporating Michigan themes and the comment prompt to input birthplaces, ad sets D1-D2 aimed to promote social connection. Incorporating a hashtag and photos sharing genuine public perspectives about biobanking, we developed ad set D3 to generate conversation.

The video ads are included in Multimedia Appendices 1 and 2; full photo albums can be viewed at facebook.com/mybloodspot.

### Target Audience

We focused our campaign on Michigan Facebook users aged 18-64 years. For all but one ad type (A1), we ran ads separately to 3 age groups: 18-30, 31-44, and 45-64 years, differentiating likely biobank participants and their peers from groups likely to include parents of young or adult biobank participants. Content was the same or very similar across age groups, but some language was tailored to address parents and participants specifically (eg, “Where were you spotted?” vs “Where was your kid spotted?”). The “page like” ad (A1) targeted 2 groups: Michigan Facebook users aged 18-64 years generally and those with an “interest” in “parenting, motherhood, or fatherhood.”

### Budget and Timeline

We set a timeline for a US $15,000, 11-week campaign to allow us to investigate the 8 ad sets using 4 ad types. Whereas “page like” ads (A1) ran for all 11 weeks, all others ran for 1 to 3 weeks. Overall, we budgeted US $5625 on ads promoting clicks to our website; US $4125 on page like ads; US $3750 on promotion of videos; and US $1500 on “page post engagement” ads.

We spent the most on the ad type promoting website clicks because the site (mybloodspot.org) has more high-quality information on the newborn screening and BioTrust topics than could be conveyed in a single ad. We also dedicated a substantial portion of our budget to page like ads. “Likes” establish a base of followers who will see future page posts, help establish credibility, and increase viral exposure as friends see one another’s likes. At a threshold of 1000, they also allow communicators to view summary statistics reporting demographics and characteristics of fans provided by Facebook’s “Audience Insights” tool.

Planning and implementing the campaign took about 4 months total, requiring weekly team meetings and approximately 20 hours per week time investment from a dedicated staff person (the lead author) with prior experience in community and social media engagement on the Michigan BioTrust to design the communications plan, create ads and wall posts, run the campaigns, and moderate discussions. The staff person created ad content and the website before the campaign; while ads ran, she posted content to the page 1-3 times weekly and moderated discussions, posting 72 comments primarily to respond to questions and concerns. Once the content and communications plan were established, running the campaign took approximately 5-10 hours per week (1-2 hours per day).

### Data Collection and Analysis to Evaluate Engagement

Facebook’s Ads Manager provides descriptive statistics from our campaign, including aggregated data reports on “General Metrics,” “Facebook Page Actions,” and “Video Actions.” These reports present metrics on performance and engagement outcomes such as reach, likes, and shares. The first 3 authors manually coded comments to distinguish those that established a social connection (eg, they tagged friends, identified their birthplace) from those that engaged in conversation about newborn screening or biobanking (eg, posting questions, opinions, feelings, personal experiences). Coders also recorded patterns of interaction: frequency of posts per user, length of discussion threads, and the number of participants in discussion threads. To examine themes of user discussion in greater detail, the authors conducted additional content analysis using ATLAS.ti, but these results will be reported in a future manuscript.

Upon consultation with the University of Michigan Institutional Review Board (IRB), this research project was deemed exempt from IRB oversight for human research participant protections.

## Results

### Overall Campaign Performance

The results of the Facebook advertising campaign overall are presented in Table 1. The campaign reached 1.88 million and led to 9186 page likes, 452 shares, and 642 comments. The average CPC in this campaign was US $0.17, and the CPM was US $0.21.

### Level 1: Observation (Red)

The reach of a Facebook ad or campaign is determined not only by ad performance but also by the target audience, the bid, and competition with other ads in the marketplace. In this campaign, 7 ad sets ran with a CPC bid type, each reaching between 87,680 and 345,587 Facebook users. Using 1 ad set with a CPM bid type (B2), we reached 1.2 million Facebook users over 3 weeks.

**Table 1:** Facebook ad campaign performance

ID^a^ Objective & Ad set	Budget, US $	Schedule	Reach	CTR^b^, %	Page likes	Web clicks	Video views	PPE^e^	Shares	Comments
A1 Page likes	$4125	Weeks 1-11	146,179	3.57	9009					
B1 Web clicks: CPC^c^ bid	$2250	Week 4	345,587	3.77	30	9563			267	99
B2 Web clicks: CPM^d^ bid	$3500	Weeks 9-11	1.2 million	0.13	8	6395			25	1
C1 Video views: newborn screening	$1500	Week 3	134,521	0.87	23		85,283		17	—
C2 Video views: biobanking	$2250	Weeks 7-8	302,768	2.27	32		146,197		102	14
D1 Page post engagement: map	$375	Week 5	119,266	10.92	22			12,108	41	374
D2 Photo album engagement: “Where were you spotted?”	$375	Week 5	87,680	11.55	40			7445	—	130
D3 Photo album engagement: posterboard perspectives	$750	Week 6	173,121	9.41	22			16,413	—	24
*Totals*	$15,000	(All)	1.88 million		9186	15,958	231,480	35,966	452	642

^a^ID: identifier

^b^CTR: click-through rate

^c^CPC: cost per click

^c^CPM: cost per 1000 impressions

^e^PPE: page post engagement

The overall population of Michigan aged 18-64 years is 6.2 million [21]. Facebook identifies the size of our target audience—Michigan Facebook users aged 18-64 years—as 5.2 million. Our campaign reached 36% of this audience (1.88 million individuals). Demographic information about those individuals reached is limited to sex and age. Of individuals reached, 57% (~1.07 million) were female; Michigan’s population is 51% female. We also reached a disproportionately young population (57% reached were aged 18-34 years; see Table 2).

**Table 2:** Demographic insights into a Facebook ad campaign

Demographic variable^a^	Michigan FB^c^ users 18-64 years Audience N=5.3 million %	Michigan FB users 18-64 years Ad reach N=1.88 million %	Michigan FB users 18-64 years Page likes N=~9200^d^ %	Michigan population^b^ (all ages) N=9.9 million %
**Race/ethnicity**				
African American	13	NR^e^	~17	14
Hispanic (all)	4	NR	~16	4
All other	83	NR	~67	82
**Sex**				
Female	53	57	~70	51
Male	45	42	~30	49
**Age, years**				
18-64	100	100	~100	63
18-24	23	28	~13	10
25-34	26	29	~24	12
35-44	19	22	~20	13
45-64	30	20	~41	28
**Household income**				
Less than US $50 K	70	NR	NR	51
US $50-$100 K	20	NR	~26	30
More than US $100 K	12	NR	NR (n<1000)	19
**Families**				
Child in home	NR	NR	~33	29
Parents (all)	32	NR	~71	NR
**Education**				
Less than diploma	NR	NR	NR	11
High school diploma	23	NR	~29	30
Some college	NR	NR	NR	24
Bachelor’s or associate’s degree	42	NR	~58	24
Advanced degree	5	NR	~12	10

^a^The table presents results from a Facebook ad campaign raising awareness about Michigan’s newborn screening and biobanking programs among Michigan Facebook users aged 18-64 years. Facebook’s ad creation tool is the source of information on the Facebook audience, Michigan Facebook users aged 18-64 years. Facebook’s ad management tool breaks down ad campaign reach by sex and age groupings. All figures are rounded.

^b^Michigan population source: US Census Bureau – State and County QuickFacts. Data derived from Population Estimates, American Community Survey, Census of Population and Housing, State and County Housing Unit Estimates, County Business Patterns, Nonemployer Statistics, Economic Census, Survey of Business Owners, Building Permits. Last revised: Tuesday, December 1, 2015, 16:11:42 EST.

^c^FB: Facebook

^d^Facebook’s “Audience Insights” tool provides information about monthly active Facebook users who have liked a page, provided that the population of a given category is greater than 1000. The N and percentages in this category are presented as estimates because the N is variable over time, owing to changes in user activity.

^e^NR: not reported.

### Level 2: Likes (Orange)

We ran a “page like” ad (A1) throughout the 11-week campaign. In Figure 2, we show just one example of the 8 photographs submitted as potential permutations of the ad set; Facebook selected, monitored, tested, and ultimately optimized the reach of the most successful images to get the most page likes. At a cost of US $4125, the “page like” ad set garnered 9009 page likes, accounting for 98% of the 9186 page likes elicited by the ad campaign.

Ads elicited 1803 “post likes” as users liked advertised content (eg, videos or boosted posts). Page post engagement ads (D1-3) generated the most and least expensive post likes in this campaign (754).

Facebook’s “Audience Insights” tool suggests that the people who liked our page skewed heavily female: approximately 70% of those who liked versus 57% of those reached. Parents, Hispanic, and African American Facebook users were also heavily represented among those who liked our page. Divided into 4 age groups, the oldest demographic (45-64 years) had the highest proportion of likes (see Table 2).

### Level 3: Exploration (Yellow)

Website click ads (B1-2), video ads (C1-2), and photo albums (D2-3) were created to facilitate deeper exploration and learning. Facebook users could engage with Web content, videos, and photo albums that included descriptions and public opinions of biobanking and newborn screening. Website click ads led to 15,958 clicks through to our website, at a cost of US $0.35 per website click. The website click ad that incurred CPM had a much lower click-through rate (CTR) than the same ad that incurred CPC: 0.13%, compared with 3.77%.

The campaign yielded 231,480 video views, a Facebook metric defined by video views lasting 3 seconds or more. On average, users watched 27.6% of the videos, with a total of 12,909 watching the videos from start to finish, at a cost of US $0.29 per 100% video view. Younger viewers were most responsive to video ads. Facebook reports “relevance scores” for individual ads, a metric on a 1-10 scale that factors in positive and negative user responses. The newborn screening and biobanking ads had relevance scores of 2 and 7, respectively, among older users, compared with 5 and 8, respectively, among users aged 18-30 years.

Photo album sets running for 2 weeks of the campaign elicited 23,858 “page post engagements.”

### Level 4: Connection (Green)

Overall, our campaign’s Facebook ads were shared 452 times. Website click ad B1 prompted 59% of all shares (267); this ad ran for 1 week and also elicited 99 comments. “Shares” both broadcast and potentially help legitimize content; health messages may be perceived as more relevant when they are mediated through friends and personal contacts [22]. Shares also drive up an ad’s organic reach. Shares of an ad in set B1, for example, pushed its organic (unpaid) reach to 22,272.

The biobank video ran for 2 weeks and prompted 102 shares.

Page post engagement ads (D1-3) were especially effective for establishing “connection” and had “relevance scores” across all age groups of 9 or 10. The map ad consisted of a graphic image of Michigan and encouraged users to write their (or their child’s) place of birth in the comments. This ad ran 1 week for only US $375. It was shared 41 times and also garnered 374 comments responding to the prompt “where were you spotted?” to build social connection. The “Where Were you Spotted?” photo album also contained a prompt for Facebook users to enter their or their child’s birthplace in the comments. This ad generated 130 comments, 80% of which responded to the prompt, while 20% engaged in conversation about biobanking or newborn screening.

### Level 5: Conversation (Blue)

Overall, 659 unique Facebook users contributed 709 comments during the ad campaign, with 642 commenting on ads directly. The vast majority of users posted 1 comment (92%), while 6.7% commented twice and 1% commented 3 to 6 times.

Discussion peaked in weeks 4-8 of the campaign, primarily in response to a Web click ad (B1, 99 comments); the photo albums (D2, 130 comments; D3, 24 comments); and the biobank video ad (C2, 14 comments).

Overall, 176 comments from 127 unique users engaged with the topics of biobanking and newborn screening. Comments included questions about consent, legality, research uses, and privacy rights; personal experiences with newborn screening; feelings of support and opposition; and intentions to opt out or continue participating in the BioTrust.

Comment threads related to public health topics ranged from 2 to 19 comments in length; among 55 threads, 62% were 2-3 comments long; 29% were 4-7 comments long, and 9% were 8-19 comments long. The campaign moderator contributed 15 wall posts and responded to comments 72 times, primarily to address questions and concerns.

Activity on the Facebook page decreased dramatically once advertising stopped, suggesting that ads were critical to both stimulating and maintaining user engagement.

## Discussion

The campaign reached 1.88 million Facebook users, yielding a range of engagement outcomes across ad sets that varied by objective, content, budget, duration, and bid type, at a relatively low cost. The page promotion, Web click, and video ads achieved strong results aligned with their objectives: 9009 page likes (US $4125), 15,958 web clicks (US $5578), and 12,909 video views to 100% (US $3750), respectively. “Boosted posts” yielded 528 comments and 35,966 page post engagements (US $1500), with high response rates to comment prompts associated with the “Where were you spotted?” campaign tagline. Overall, the ads led to 452 shares and 642 comments, including 176 discussing newborn screening and biobanking.

As a field, public health has been called to task for underutilizing the value of harnessing the social characteristics of social networking sites to advance the social service of informing, educating, and empowering the public [1,2,17,20,22-25].

Low-level, one-way engagement has been identified as “the stage of engagement where most social media efforts in public health and health promotion languish or terminate” [16]. Dynamic, dialogic approaches open opportunities for the public to share content, ask questions, contribute, advocate, respond, and interact with a community of Facebook users. Public health communicators can in turn listen and respond, adapt messages, answer questions, and gauge and consider public sentiment. In this paper, we have presented a campaign that aimed to achieve engagement goals across 5 stages of engagement—reach, likes, exploration, connection, and conversation—and we have examined the results in a framework that aligns these results with Facebook advertising metrics and situates them in both context and process.

The campaign reached a very large number of individuals (1.88 million Facebook users), especially for a short-term campaign. Specifically, we reached 36% of our potential ~5.2 million Facebook user audience. More examples in the literature would be helpful to determine the typical proportional reach of Facebook ads targeting large populations, and the relative effects of time, budget, and ad settings on maximizing reach among a broad population. In this study, running an ad that incurred cost based on impressions rather than clicks significantly boosted the overall reach of the campaign. Further, overall, funding the campaign allowed us to build our audience and push information out that would not have otherwise been accessible to users.

The campaign’s Facebook page ended the campaign with 9186 “likes” and the page promotion ad alone established a fan base in 11 weeks that surpassed those of related but much more well-known and established entities. For example, at the time, the University of Michigan School of Public Health had 7125 likes [26] and the Michigan Department of Health and Human Services had 8572 likes [27]. For further context, we did not approach the fan base of Shakira, with 104 million likes [28].

Other metrics often applied in evaluating Facebook ad performance are CTRs, CPC, and CPM. Benchmark CTRs vary widely by industry and ad type, but a Facebook ad sales representative suggested the goal of 3.0% for this 2015 campaign. Average metrics for nonprofits, according to a 2013 industry publication [29], were 0.21% CTR, US $0.19 CPC, and US $0.52 CPM. In a public health study of the effectiveness of Facebook ads for recruiting survey participants on young adult substance use, a 2011 campaign that cost US $6628 over 13 months reported a CTR of 0.05% and cost US $0.45 per click and US $0.35 per 1000 impressions [6]. Our campaign met or exceeded these values. The average CTR for ads in this campaign optimized for clicks was 4.54%. Page post engagement ads yielded CTRs of 9.41% to 11.5%. The average CPC was US $0.17, and the CPM was US $0.21.

The spectrum framework that we use incorporates metrics such as “clicks” (CTR, CPM, CPC) and “likes” that are readily available on the Facebook platform, and also includes “conversation” that can be analyzed quantitatively and qualitatively to achieve more robust and meaningful evaluation. In this campaign, Web click, video, and page post engagement ads prompted users to explore and discuss public health topics, creating an open and transparent forum for education and discussion of newborn screening and biobanking. Users posted 176 comments on these topics and engaged in 53 relevant discussion threads during the campaign. These patterns of interaction are indicative of participatory learning [19].

These outcomes indicate the potential for social media networking sites to innovate public health communications. Notably, however, raising awareness about Michigan’s biobank initiative among its large population of participants and parents presented challenges and risks. The information was unfamiliar, complex, and sensitive. Thus, our campaign needed to present opportunities for users to explore information deeply and to interact, both with our organization and with peers. We used Facebook ads because they had the potential to reach a large audience quickly and economically and facilitate transparent discussion. The content, context, and tone of our awareness campaign were presumably important factors determining interest and engagement among users. The peculiar story of the Michigan BioTrust meant that the relevance of the information we delivered depended on the age and birthplace of the users and children of the users we reached, that conversation was likely sparked by diverging opinions and levels of comfort with the initiative, and that low levels of awareness likely made the information more difficult to deliver and digest.

Factors that have been found to affect engagement outcomes in the context of health promotion on social networking sites include privacy and stigma concerns associated with particular conditions (eg, sexually transmitted diseases, diarrhea); the purpose and utility of a Facebook page or organization (eg, support groups vs public awareness); and the context and content of subject matter [30-32]. Standardized metrics, well-defined terms, attention to context, and detailed reporting of processes will help health researchers to compare outcomes of social media engagement. The prism framework used here (Figure 1) might influence the way communicators plan and analyze the use of Facebook in health education and promotion. Communicators can strategically target engagement outcomes across the spectrum using variable ad types (objectives) and fostering user interaction. Low-level engagement can be valuable in itself or to stimulate higher-level engagement. Active “exploration” can be shallow or deep (eg, a photo view vs extensive reading) but the messaging is still one-way; the next step is to stimulate high-level, multi-way engagement to take advantage of the social aspects that distinguish social networking sites from traditional media [2,18,21-23] and to address the need for more evidence on approaches to stimulating interaction [15,16].

### Limitations and Future Studies

Understanding Facebook as a communications platform is a time-intensive endeavor, and the lack of control over algorithms and certain organizational features can be challenging for public health communicators and researchers. A limitation to public health communication via Facebook is that the representativeness of the audience targeted and reached cannot be fully determined through typical epidemiological methods because of Facebook’s restrictions on its release of population demographics. However, as a Facebook campaign begins to reach large fractions of the total population, the confidence that the campaign reached the full range of diversity in the population increases. Social networking analysis can provide insight into engagement and its social drivers, but was beyond the scope of this study. More research needs to be done on the dynamics of population-based communications using Facebook and its advertising infrastructure. This campaign ran for a relatively short time span (11 weeks), with minimal Facebook activity before and after the campaign; results might vary for an organization with a more steady, established, and ongoing social media presence. More studies are needed to test specifically how input variables (eg, ad types, bid type) affect engagement outcomes.

### Conclusions

The presented case suggests public health communicators can use Facebook ads to spur actions and interactions with users across a spectrum of user engagement by creating ads with diverse ad types and multiple engagement goals. A wide set of inputs filtered through the “prism” of Facebook’s algorithms for ad delivery yields an entire spectrum of engagement outcomes. Our results, mapped onto this spectrum of Facebook actions, indicate potential for social networking sites to bring the public into public health communications.

Facebook advertising campaigns can efficiently reach large populations and achieve a range of engagement outcomes by diversifying ad types, bid types, and content. This campaign provided a population-based approach to communication that also increased transparency on a sensitive and complex topic by creating a forum for multi-way interaction. By offering a platform on which individual citizens can access professionals and peers in an open forum, Facebook is a promising tool for meeting the arising objectives of personalized public health.

## Appendix

Multimedia Appendix 1 Facebook video ad to raise statewide awareness of Michigan's newborn screening program.

Multimedia Appendix 2 Facebook video ad to raise statewide awareness of Michigan's large population biobank.

## Abbreviations

BioTrust: Michigan BioTrust for Health
CPC: cost per click
CPM: cost per 1000 impressions
CTR: click-through rate
IRB: Institutional Review Board
